# Medical students’ attitudes towards early clinical exposure in Iran

**DOI:** 10.5116/ijme.5749.78af

**Published:** 2016-06-19

**Authors:** Mahboobeh Khabaz Mafinejad, Azim Mirzazadeh, Soheil Peiman, Nasim Khajavirad, Mojgan Mirabdolhagh Hazaveh, Maryam Edalatifard, Seyed-Farshad Allameh, Neda Naderi, Morteza Foroumandi, Ali Afshari, Fariba Asghari

**Affiliations:** 1 Department of Medical Education, Tehran University of Medical Sciences, Tehran, Iran; 2 Department of Internal Medicine, Tehran University of Medical Sciences, Tehran, Iran; 3 Department of Medical Ethics, Tehran University of Medical Sciences, Tehran, Iran

**Keywords:** Early clinical exposure, reflection, undergraduate medical curriculum

## Abstract

**Objectives:**

This study was carried out to investigate the medical students’ attitudes towards early clinical exposure at Tehran University of Medical Sciences.

**Methods:**

A cross-sectional study was conducted during 2012-2015. A convenience sample of 298 first- and second-year students, enrolled in the undergraduate medical curriculum, participated in an early clinical exposure program. To collect data from medical students, a questionnaire consisting of open-ended questions and structured questions, rated on a five-point Likert scale, was used to investigate students’ attitudes toward early clinical exposure.

**Results:**

Of the 298 medical students, 216 (72%) completed the questionnaires. The results demonstrated that medical students had a positive attitude toward early clinical exposure. Most students (80.1%) stated that early clinical exposure could familiarize them with the role of basic sciences knowledge in medicine and how to apply this knowledge in clinical settings. Moreover, 84.5% of them believed that early clinical exposure increased their interest in medicine and encouraged them to read more. Furthermore, content analysis of the students’ responses uncovered three main themes of early clinical exposure, were considered helpful to improve learning: “integration of theory and practice”, “interaction with others and professional development” and “desire and motivation for learning medicine”.

**Conclusions:**

Medical students found their first experience with clinical setting valuable. Providing clinical exposure in the initial years of medical curricula and teaching the application of basic sciences knowledge in clinical practice can enhance students’ understanding of the role they will play in the future as a physician.

## Introduction

The initial years of medical education are critical to form the pre-conceived attitudes of medical students towards medicine and to familiarize them with the roles they will play in the future as a physician.^1^ Accordingly, along with the drive toward the reforms of medical curricula, there has been a growing consideration to provide some opportunities for integration of pre-clinical and clinical phases.^2^^,^^3^ Many medical schools around the world, in response to these needs, implement various types of vertically and horizontally integrated practical experience into the early years of curriculum to introduce important issues in medicine to pre-clinical medical students.^4^^-^^7^ Early clinical exposure (ECE) is a way to integrate the knowledge of basic and clinical sciences and the psychosocial aspects of medical practice.^8^ There is some evidence showing that delivery of ECE programs in the early years of medical curricula may move medical education towards the real context of practice. They facilitate medical students’ transition to the clinical phase, help them develop a professional identity, increase their motivation and satisfaction and make them more aware of the application of basic sciences in medical practice, and boost their confidence to handle the patients’ problems in practice.^1^^, ^^9^^-^^11^

Iranian medical universities have delivered a traditional Flexnerian undergraduate medical education program for a long period. Following the recommendations of the Iranian Ministry of Health and Medical Education (MHME) and a need to respond to the profound contextual changes, the undergraduate medical curricula in Iran are changing.^12^ Parallel to the reform in undergraduate medical curricula, Tehran University of Medical Sciences (TUMS) introduced a renewed curriculum emphasizing aspects such as vertical and horizontal integration, early clinical and community exposure, case-based discussion and team-based learning.^13^ Delivery of the ECE program during the pre-clinical phase is one of these changes.

Although the value of ECE in medical education is well-established,^14^^,^^15^ studies have shown that assessing the perceptions of medical students about their first clinical exposure can be helpful in forming the physicians’ professional practice. Nevertheless, little attention has been paid to the student perceptions of early clinical exposure in Iran. The purpose of this study, therefore, was to examine the pre-clinical students’ attitudes towards the ECE program.

## Methods

### Study design

A cross-sectional study was conducted at TUMS, school of medicine, one of Iran's largest and oldest medical schools. The study was performed at the Internal Medicine ward, a tertiary care academic center, during 2012-2015.

### Participants

The initial convenience sample included 298 first- and second-year medical students who participated in the ECE program in groups of 10. Of these, 216 (72%) completed the questionnaire at the end of the program. The sample comprised of 46% females, in the age range 21-18.5 years. Tehran University of Medical Sciences Ethics Committee approved the study. Participation was voluntary and anonymous, and no incentives were provided.

### Data collection method

Data were collected using a researcher-developed questionnaire, including three open-ended questions and seven structured questions, rated on a five-point Likert scale (strongly agree, agree, neutral, disagree, and strongly disagree). Our methodology to develop the questionnaire consisted of several steps. First, the items were generated based on a critical review of the literature and informal interviews with the clinical faculty involved in teaching the medical students. Then, the questionnaire was piloted on a sample of 20 medical students to ensure its clarity. At that point, the questionnaire was tested for validity by medical education and clinical experts. The reliability of the questionnaire was acceptable, with a Cronbach’s alpha of 0.72.

### Procedure

In this descriptive study, a weekly early clinical exposure was organized for medical students in the pre-clinical phase in the internal medicine ward. The main aim of the ECE program was to provide an opportunity for exposure to the real clinical setting in the initial years of the curriculum. The length of ECE program was 5 hours for each group. One week before the program, each group of students was informed about the rules of participation in the program and was given the clinical case which had been introduced in grand rounds via a website. The ECE program at TUMS involved multiple events, including a brief description of the objectives and responsibilities of a medical student towards the patients, familiarity with the regulations of internal medicine ward, participation in the grand rounds, attendance at the bedside, and reflection sessions. The ground round was attended by pre-clinical and clinical students, residents, and clinical and basic sciences faculty members. During the session, the faculty members and students discussed each clinical case. The discussion included patient history, chief complaints, symptoms, results of diagnostic methods and treatment in brief. Clinical cases were presented in a step-by-step manner to challenge the reasoning of learners. Students were also asked some basic sciences questions related to the cases to stimulate them to apply their basic sciences knowledge in solving the patient’s problems. After the round, some patients were selected to be visited by the medical students under the supervision of a clinical faculty member. At the end of the program, an opportunity was provided for the students to complete the questionnaire and to reflect on their first clinical experience and express their perceptions.

### Data analysis

Statistical analysis was performed using SPSS software (version 20). The quantitative descriptive analysis was used to evaluate the students’ attitudes and content analysis to analyze the notes about their first experiences. Furthermore, differences in attitudes towards ECE between male and female students were analysed with the Mann-Whitney test. A P value of 0.05 was used to determine significance.

## Results

Based on the quantitative data, the majority of students (80.1%) stated that the ECE program could familiarize them with the role of basic sciences knowledge in medicine and the way to apply it in clinical settings. Further, 84.5% of them believed that the early clinical experience increased their interest in medicine and motivated them to read more. Also, 87.3% of students mentioned that group discussion during the grand round could help them to reflect on their experiences and share them with others. Furthermore, 89.1% of students agreed (completely agree/agree) with the usefulness of the grand round, and 8.9% were neutral. In general, most students (89.7%) completely agreed/agreed that participation in ECE was a good experience and contended they were eager to attend such programs more. The descriptive analysis of the questionnaire is presented in Table 1.  As presented in Table 2, no significant differences in any of the items were observed between gender (male and female) and academic years (first and second- year).

**Table 1:** Students’ views towards early clinical exposure

Items	Strongly agree %	Agree %	Neutral %	Disagree %	Strongly disagree %
Familiarity with the role of basic sciences knowledge in clinical settings	35.9	44.2	15	3.9	1
Familiarity with doctoring skills in medicine	33.2	42.5	12.2	8.7	3.3
Motivation toward learning more	53.4	31.1	12.1	2.9	0.5
Familiarity with the roles and responsibilities of clinical students	39.1	20.3	20.6	15.9	4.1
Usefulness of participation in ground rounds	55.6	33.5	8.9	1.5	0.5
Providing opportunities to discuss and share knowledge	21.1	66.2	10.7	1	1
Utility of early clinical exposure and providing more experiences	49.5	40.2	7.8	1.5	1

At the end of the questionnaire, three open-ended questions were provided for students to reflect on any aspect of the early clinical exposure. Content analysis of the students’ responses uncovered three main themes of early clinical exposure, were considered helpful to improve learning: “integration of theory and practice”, “interaction with others and professional development” and “desire and motivation for learning medicine”.

**Table 2:** Students’ views towards early clinical exposure, by gender and academic years

Items	Gender	Academic years
Familiarity with the role of basic sciences knowledge in clinical settings	0.52	0.92
Familiarity with doctoring skills in medicine	0.60	0.44
Motivation toward learning more	0.68	0.46
Familiarity with the roles and responsibilities of clinical students	0.97	0.36
Usefulness of participation in ground rounds	0.51	0.14
Providing opportunities to discuss and share knowledge	0.33	0.96
Utility of early clinical exposure and providing more experiences	0.77	0.95

Most students reported that the ECE program enhanced their understanding of basic sciences knowledge and helped them to integrate it into clinical cases. One participant said that:

“I'm constantly thinking how I have applied the lessons learned and how I can link different lessons in basic sciences, so that if I encounter clinical cases I can solve them based on basic sciences knowledge.” (Male second-year student)

Another reported that:

“During wardattendance, I remember the information obtained, which was sometimes incomplete, and I tried to find a reason for the disease through basic sciences knowledge.” (Male first-year student)

However, some students stated understanding clinical cases using only basic sciences was difficult, but overall, most agreed to find the application of basic sciences an enjoyable experience.

“Clinical cases are very complicated… some of the topics discussed at grand rounds were not understood.” (Female first-year student)

From the participants’ viewpoint, they gained more satisfaction via interaction with other students, clinical faculty members and patients during the program. One of the students mentioned several points in this regard:

“Observing the patients closely, reviewing different orders by teachers and clinical students and discussing which investigation is more suitable for the treatment of diseases ... in fact, being a physician is completely tangible in practice.” (Female second-year student)

Some students stated they would better understand the roles of doctors in delivering effective services to patients via attending the shared programs. One participant said:

“By observing the interactions and events in hospital, I could have a clear picture of the duties of a physician, though lack of necessary information to pursue professional debates was a little disappointing!!” (Male first-year student)

According to the participants of this study, the motivation towards medicine was stronger after visiting the patients and attending their bedside.

“The first time at the hospital, not as a patient… however, as a physician, I felt not until then that I am a doctor.” (Male second-year student)

The study also revealed that most students had a desire to attend more ECE events. The most stated demand of students was more participation in such programs. They contended that the ECE program would be helpful if it were continued in each organ system block.

The following comments by some students show how they described their attitude on the usefulness of the ECE program:

“This experience made me more focused. I study my lessons more meaningfully and make sure that all of this information is required in future.” (Male second-year student)

“To me, this experience is necessary for students of Basic Sciences.” (Female second-year student)

## Discussion

Earlier studies have focused on the existing types of early clinical exposure in medical education,^6^  Hence, the purpose of this study was to assess the medical students’ attitudes and to explore their perceptions of early clinical exposure.

The majority of medical students believed that ECE could strengthen their learning in the pre-clinical phase. The students’ views during the program indicated that ECE was a valuable learning experience for them. It seems possible that these results were because ECE helped students to appreciate the application of basic sciences knowledge in clinical problem-solving, to communicate the relevance of basic sciences to their future practice and to prepare them for the transition to clinical settings. These findings are in agreement with Hampshire’s (1998) results, which showed early experience had helped medical students to understand basic medical sciences.^16^ Dahle et al. recommended that vertical integration of basic sciences and clinical medicine could contribute to a better understanding of important biomedical principles.^4^ Also, this study revealed that students viewed the early clinical experience as an opportunity to familiarize them with doctoring skills of patient care.

Another important finding in this study was that the ECE program increased students’ interest in being a doctor. It seems that interacting with other clinical students, clinical faculties, physicians and patients motivated them and provided them with an opportunity to discuss and share their knowledge. Moreover, it might be that these students benefited from familiarity with the physicians’ expected roles and responsibilities they should do in the future. Dyrbye et al. believed that interactions with and between peers and faculty are also important in professional development.^17^ The present findings seem to be consistent with those of other researchers.^16^^,^^18^

This study indicated how ECE program could be used as a learning experience to promote the positive reaction of freshman medical students toward learning in the pre-clinical phase. Our research demonstrated that freshman medical students’ attending the ECE program was associated with their increased satisfaction, which corroborates these earlier findings.^19^ Several studies support the positive impact of ECE program on medical students’ satisfaction with medical curricula. Duque et al. (2003) reported that the medical students who had early clinical experience were usually more satisfied with their medical education.^20^ Sathishkumar et al. (2007) reported that early exposure in endocrine physiology course in the first year of medical education was a valuable event, and medical students clearly enjoyed this experience.^21^

### Limitations and strengths

The strength of this study was the high volume of samples that were exposed to clinical settings in the initial years of medical education. This study was the first one that was conducted at TUMS to assess the medical students’ attitudes towards early clinical exposure, where integration has a great importance. The major limitation of this study was that the students’ attitudes were evaluated immediately after participation in the early clinical exposure. Hence, the long-term effects of this experience on the students’ performance and attitude during the clinical years should be assessed. Furthermore, ignoring the students’ incentive may have influenced the response rate and have caused the unwillingness of some medical students to complete the questionnaire. However, the questionnaire was distributed by the secretary of internal medicine ward, who informed the medical students that their responses would not affect their participation. Moreover, some cases that were presented in the grand rounds were very complex for pre-clinical students, which might have influenced the students’ attitudes. This study provided a framework for curriculum planners to integrate the basic and clinical sciences in the initial years of a revised medical curriculum.

## Conclusions

Medical students found their first experience with clinical settings valuable and rewarding. Providing clinical exposure in the first years of medical education and presenting the application of basic sciences knowledge in the clinical practice can enhance students’ motivation and understanding of the role they will play in the future as a physician. The ECE program was shown to have the potential benefits to guide the policymakers and other stakeholders to provide a framework for the integration of basic and clinical sciences. 

### Acknowledgements

The authors thank all medical students who participated in this program as well as the clinical faculty members and Ms. Kabiri in the Division of General Internal Medicine of Imam Khomeini Hospital for their support in implementing the program.

### Conflict of Interest

The authors declare that they have no conflict of interest.

## References

[1] Littlewood S, Ypinazar V, Margolis SA, Scherpbier A, Spencer J, Dornan T (2005). Early practical experience and the social responsiveness of clinical education: systematic review.. BMJ.

[2] Spencer AL, Brosenitsch T, Levine AS, Kanter SL (2008). Back to the basic sciences: an innovative approach to teaching senior medical students how best to integrate basic science and clinical medicine.. Acad Med.

[3] Vidic B, Weitlauf HM (2002). Horizontal and vertical integration of academic disciplines in the medical school curriculum.. Clin Anat.

[4] Dahle LO, Brynhildsen J, Behrbohm Fallsberg M, Rundquist I, Hammar M (2002). Pros and cons of vertical integration between clinical medicine and basic science within a problem-based undergraduate medical curriculum: examples and experiences from Linköping, Sweden.. Med Teach.

[5] Dornan T, Bundy C (2004). What can experience add to early medical education? Consensus survey.. BMJ.

[6] Başak O, Yaphe J, Spiegel W, Wilm S, Carelli F, Metsemakers JF (2009). Early clinical exposure in medical curricula across Europe: an overview.. Eur J Gen Pract.

[7] Dornan T, Littlewood S, Margolis SA, Scherpbier A, Spencer J, Ypinazar V (2006). How can experience in clinical and community settings contribute to early medical education? A BEME systematic review. Med Teach.

[8] Dandekar K. The impact of early clinical exposure on first MBBS students. International Journal of Healthcare and Biomedical Research. 2014;2(4):176-81.

[9] Krajic Kachur E (2003). Observation during early clinical exposure - an effective instructional tool or a bore?. Med Educ.

[10] Wenrich MD, Jackson MB, Wolfhagen I, Ramsey PG, Scherpbier AJ (2013). What are the benefits of early patient contact?--A comparison of three preclinical patient contact settings.. BMC Med Educ.

[11] Diemers AD, Dolmans DH, Verwijnen MG, Heineman E, Scherpbier AJ (2008). Students' opinions about the effects of preclinical patient contacts on their learning.. Adv Health Sci Educ Theory Pract.

[12] Hamilton J. The renewal of medical education in Iran: progress and challenge. Med J Islam Repub Iran.2011;25(2): 53-6.

[13] Mirzazadeh A, Mortaz Hejri S, Jalili M, Asghari F, Labaf A, Sedaghat Siyahkal M, Afshari A, Saleh N (2014). Defining a competency framework: the first step toward competency-based medical education.. Acta Med Iran.

[14] Shirzad H, Moezzi M, Khadivi R, Sadeghi B, Madhkhan A. Effect of early clinical exposure on attitude and performance of first year medical students. Journal of Shahrekord University of Medical Sciences. 2011;13(1):69-78.

[15] Prithishkumar IJ, Holla SJ. Early clinical exposure as a teaching learning tool to teach neuroanatomy for first year occupational and physical therapy students-our preliminary experience. Indian J Physiother Occup Ther. 2012;6(2):59-62.

[16] Hampshire AJ (1998). Providing early clinical experience in primary care.. Med Educ.

[17] Dyrbye LN, Harris I, Rohren CH (2007). Early clinical experiences from students' perspectives: a qualitative study of narratives.. Acad Med.

[18] Rooks L, Watson RT, Harris JO (2001). A primary care preceptorship for first-year medical students coordinated by an Area Health Education Center program: a six-year review.. Acad Med.

[19] Abramovitch H, Shenkman L, Schlank E, Shoham S, Borkan J (2002). A tale of two exposures: a comparison of two approaches to early clinical exposure.. Educ Health (Abingdon).

[20] Duque G, Gold S, Bergman H (2003). Early clinical exposure to geriatric medicine in second-year medical school students--the McGill experience.. J Am Geriatr Soc.

[21] Sathishkumar S, Thomas N, Tharion E, Neelakantan N, Vyas R (2007). Attitude of medical students towards Early Clinical Exposure in learning endocrine physiology.. BMC Med Educ.

